# Early ontogeny social deprivation modifies future agonistic behaviour in crayfish

**DOI:** 10.1038/s41598-019-41333-8

**Published:** 2019-03-20

**Authors:** Jiří Patoka, Lukáš Kalous, Luděk Bartoš

**Affiliations:** 1 Department of Zoology and Fisheries, Faculty of Agrobiology, Food and Natural Resources, Czech University of Life Sciences Prague, Kamýcká 129, Praha - Suchdol, 165 00 Czech Republic; 2 Department of Ethology, Research Institute of Animal Production, Přátelství 815, Praha, 104 01 Czech Republic; 3 Department of Animal Science and Ethology, Faculty of Agrobiology, Food and Natural Resources, Czech University of Life Sciences Prague, Kamýcká 129, Praha - Suchdol, 165 00 Czech Republic

## Abstract

Social deprivation early in life affects further individual development and leads to irreversible behavioural alterations later in life. Although the syndrome is well-studied in vertebrates including humans, its presence in invertebrates has been described only in eusocial insects and cockroaches. Here we show the first evidence of social deprivation in subsocial decapod crustaceans, based on analysis of video-recorded agonistic encounters of juvenile red swamp crayfish (*Procambarus clarkii*, Girard). In comparison with maternally incubated juveniles, isolated crayfish had altered repertoires, numbers and frequency of agonistic interactions similar to those described in vertebrates. Our results support the view on the syndrome of social deprivation as a ubiquitous trait in species with developed maternal care across diverse taxa.

## Introduction

Psychosocial stress related with early prolonged isolation of offspring from their mother has been repeatedly shown to trigger a variety of dramatically and irreversible long-term alterations in behavioural patterns and physiological mechanisms of adaptation of deprived individuals^1,2^. Behavioural impairments make affected individuals unable to properly respond to environmental and social stimuli with impacts on exploration, foraging and feed intake, reproduction, social and agonistic interactions, communication, learning, growth, susceptibility to diseases, process of recovery, morbidity, and survivorship^3–9^.

Previous studies investigating aforementioned social deprivation shaping behaviour have focused mainly on social vertebrate species with emphasis on altricial birds^10,11^ and mammals^1,4,12–14^ including humans^15–17^. A very different situation exists in invertebrates: although many papers have focused on studies of general aggressive behaviour^18^, behavioural impairments associated with social deprivation have been studied only in eusocial insects^19–21^ and in one species of group living cockroaches^22^. Even though it was previously suggested that the social deprivation syndrome seems to be a ubiquitous trait over both vertebrate and invertebrate social species^22^, this phenomenon is still hypothesized and scarcely studied in non-eusocial invertebrates.

Compared to eusocial arthropods, subsocial crustaceans (species where parents care for their offspring for some period) live not in castes in colonies, but in most cases solitary in adult stage. Nevertheless, maternal care is developed in the early life of offspring in many species^23^. Freshwater crayfish (Decapoda: Astacidea) are a good model of subsocial crustaceans to test an influence of hypothesized social deprivation on agonistic behaviour, because juveniles stay associated with their mother longer than is necessary to be able to be free-living^24–26^. During the period of egg incubation, the ovigerous females carry eggs on abdominal appendages (pleopods) and exhibit a maternal care behaviour towards offspring: fanning pleopods, grooming, and cleaning^24^. After young crayfish have hatched, the mother inhibits their aggressiveness by production of brood pheromones^25,27^.

Considering the large diversity of sociality types in arthropods, the investigating of social deprivation, both at the physiological and the behavioural level, should bring interesting and crucial data for comparison of species and their sociality within the group of invertebrates. Therefore, we conducted bioassays to compare behavioural responses in agonistic encounters of juvenile crayfish reared in isolation from their mother to those of their conspecifics reared naturally with mother. We hypothesized a higher level of aggressiveness in early separated juveniles than in maternally incubated ones.

## Results

We recorded 28,528 dyadic encounters. Subsequently, non-aggressive interactions (Approach) were excluded (N = 19,756) and remaining agonistic encounters (N = 8,772) were used for the analysis. Juveniles which lost one or both chelae (N = 33) were excluded from the trial and the number of dyadic encounters was further reduced to 7,236. These encounters were summed according to Group type, day, and type of the interaction (Attack, Fight, and Harassment). This sum, the Total numbers of agonistic encounters, was then used as a dependent variable for the statistical analysis.

**Figure 1:**
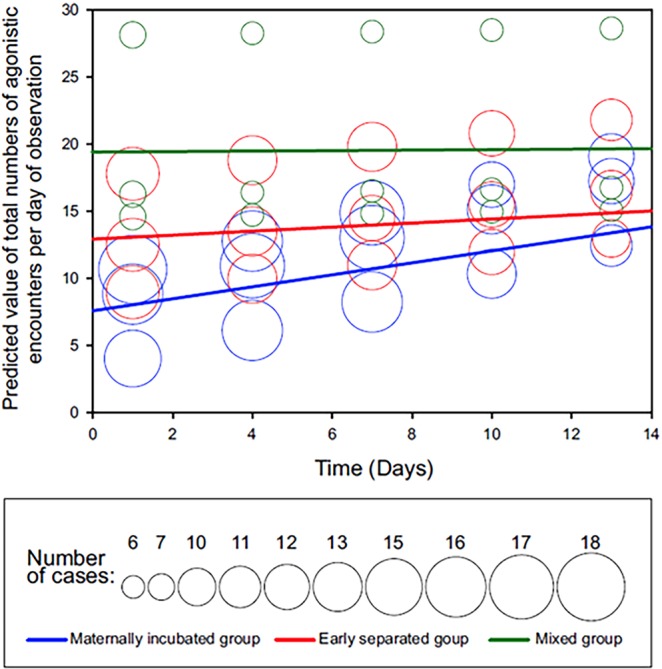
Bubble plot of predicted values of total numbers of agonistic encounters plotted against Time according to Group. The high predicted values of the total numbers of agonistic encounters per day equal to about 28 for the Mix group are not outliers. These high values were achieved by seven different individuals and occurred in each of the seven groups with the frequency between 1 and 5.

Total numbers of agonistic encounters each day of the observation within the three Group types (maternally incubated = MI, early separated from the mother = ES, mix of both of them = Mix) showed significant differences between MI-ES, MI-Mix, and ES-Mix (GLMM, F_(3, 489)_ = 6.31, P = 0.0003, Fig. 1). The time trend increased over time in MI (Solution for Fixed Effects, t = 3.97, P < 0.0001, Fig. 1), an increase was indicated in ES (t = 1.78, P = 0.076, Fig. 1), and there was no change with time in Mix (t = 0.16, NS, Fig. 1).

**Figure 2:**
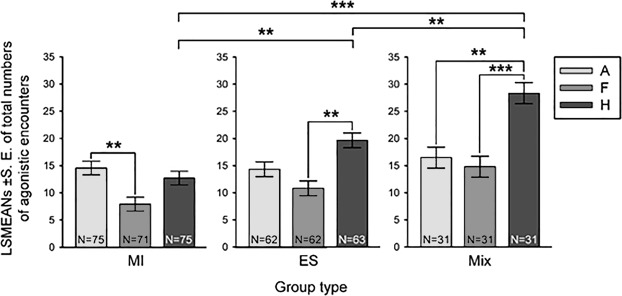
The ratio of agonistic encounters (A – Attack; F – Fight; H – Harassment) within the three Group types of juvenile crayfish: MI – maternally incubated; ES – early separated from mother; Mix – combined group of previous two origins in ratio 1:1. Each bar represents a LSMEAN value for specific Type of agonistic activity within all groups of each type. The asterisks indicate statistical differences (**P < 0.01, ***P < 0.001).

Total numbers of agonistic encounters differed among Group types (GLMM, F_(2, 489)_ = 13.05, P < 0.0001, not shown) and among Attack, Fight and Harassment (GLMM, F_(2, 489)_ = 25.63, P < 0.0001, not shown). There were significant differences in total numbers of agonistic encounters between Group types according to the Type of agonistic activity (GLMM, F_(4, 489)_ = 4.93, P = 0.0007, Fig. 2). We found no significant differences in numbers of Attacks and Fights between Group types. However, in the Harassment activity, the highest numbers were shown in Mix when compared to MI and ES; the lowest Harassment activity was found in MI (Fig. 2).

The ratio of total numbers of agonistic encounters (Attack, Fight, and Harassment) in regard to Group type (MI, ES, and Mix) after the end of observation exhibits the significant differences shown in Fig. 2: Attack was higher than Fight within MI, Harassment was higher than Fight within ES, and Harassment was higher than both Attack and Fight within Mix.

## Discussion

Based on obtained data, it was found that deprived juveniles were more active in agonistic behaviour than maternally incubated juveniles, and they preferred attacking the opponent from behind than fighting. Abnormal attack patterns associated with hyperarousal behaviour had been registered also in socially deprived vertebrates^8,28^.

Moreover, a combination of socially deprived individuals with those maternally incubated in one group resulted in developmental changes in various agonistic behavioural components and even led to worse consequences than in groups of early separated juveniles only. Specifically, these effects consisted of: (i) significantly highest total numbers of agonistic encounters with an almost unchanging trend over time; (ii) highest numbers of Harassments among all evaluated Group types, and (iii) higher numbers of Harassment interactions than Attack and Fight. If the deprived individuals were responsible for a majority of the increased Harassment behaviour in the Mix group, these socially deprived crayfish could be harmful to maternally incubated conspecifics because of disruption of normal agonistic behaviour including the higher frequency of attack from behind. The described changes in behaviour could lead to lower growth rates than in maternally incubated juveniles^29^.

Our results showed that early isolation from the mother significantly affected the behaviour of young red swamp crayfish and led to different agonistic interactions in quantity, quality, and also in progression over time. Hence, these behavioural changes compared with those of maternally incubated crayfish indicate the deprivation. The importance of social interactions between mother and offspring in this sub-social invertebrate supports the idea that isolation from the mother might impact the behaviour of offspring, irrespective of sociality, in both vertebrate and invertebrate social species^22^.

Our findings have several practical implications. Certain crayfish species including red swamp crayfish are produced in aquaculture for human consumption. One of the common techniques of their production in captivity is artificial incubation of eggs away from the mother; this method is recommended by certain authors as advantageous^30–33^. Moreover, some authors also noted captive breeding of endangered crayfish species as a useful tool in wildlife conservation^34^. In contrast, our results showed that a sufficient quantity of crayfish incubated without their mother and released into a locality with a weak population could significantly disrupt the behaviour of wild conspecifics. Therefore, this method of artificial reproduction should be avoided in a conservation strategy.

In regard to the overlap of our results into commercial breeding as well as into the management of endangered species of subsocial invertebrates, we strongly recommend our presented findings to the attention of stakeholders such as conservationists, wildlife managers and producers. We anticipate our assay to be a starting point for detailed research of individual development of subsocial invertebrates with a special focus on irreversibility of aforementioned behavioural alterations later in life of deprived crayfish.

## Methods

### Experimental design

The red swamp crayfish (*Procambarus clarkii*) was chosen as a model species to determine the existence of social deprivation syndrome in subsocial crustaceans. Three sorts of groups (further referred as Group types) were formed: (i) maternally incubated (MI): all individuals in the group were reared with mother to free-living stage; (ii) early separated (ES): all individuals in the group were isolated from mother at stage 2; (iii) mixed group (Mix): individuals were combined from both types of origin in ratio 1:1. Five individuals from three maternally incubated MI groups were killed by conspecifics and hence after the observations these groups were terminated. One Mix, three MI, and three ES groups were also terminated because of multiple tag loss of their members. Hence, 12 MI, 11 ES, and 6 Mix groups completed the experiment in its entirety. Variation in body size was equal among groups.

### Incubation and rearing

Eight berried females of the red swamp crayfish were obtained from commercial producers and placed into individual tanks (Faunarium PT-2300, 370 × 220 × 165 mm) until offspring hatched. Approximately half of the juveniles were subsequently naturally reared on mother’s pleopods (maternally incubated) and the others were stripped from mothers’ pleopods at intermoult stage 2 and reared separately depending on maternal line. Mothers, with eggs attached, were moved to grow-out tanks, after which juveniles were separated and isolated from their mother. These individuals were separated from the mother without any contact by hand or tool, only by moving the mothers’ abdomen under the water. Separated juveniles were stocked in grow-out tanks (Faunarium PT-2300) immediately after isolation from their mother. This procedure prevented any ongoing social experience of separated juveniles with their mother, contrary to the maternally incubated individuals. Maternally incubated juveniles were released into the grow-out tanks (Faunarium PT-2300) in intermoult stage 3 when they became free-living and the experiment was commenced.

### Grouping and marking

After this period, the juveniles were inspected for missing appendages and 234 individuals without visible injuries were distributed to 39 groups each of six individuals within the three Group types: MI (groups N = 18, juveniles N = 108); ES (groups N = 14, juveniles N = 84); Mix (groups N = 7, juveniles N = 42). Juveniles which had not been involved in the experiment, and adult females were returned to the supplier.

Each individual in a group was marked with a unique colour tag (one or two spots of a combination of blue, red, or yellow colour) applied by quick drying painting (Avon Speed Dry nail enamel) on the dorsal side of the cephalothorax. Subsequently, six-member groups were released into the plastic observation tanks (Faunarium PT-2255, 230 × 155 × 170 mm) with pale silica sand (grain-size 2.0 mm) on the bottom and 50 mm high water level. Tanks were filled with de-chlorinated tap water. The members of the same group were marked in one moment and released to the tank one by one in random order.

Groups were kept under constant conditions (water temperature ~24 °C, 12:12 h light: dark cycle, oxygen 5.0–7.1 mg.l^-1^, pH 6.7–7.5) and they were fed once per 24 hours to satiety with TetraMin Crisps (Tetra GmbH, Melle, Germany). The food was distributed over the entire tank bottom in order to reduce resource competition. To avoid competition for shelter, there were no objects in the tank which might be used as a shelter. Therefore, the only factor influencing competition in the experiment was space, as opposed to food. Stock density was equivalent to ~170 individuals per m^2^. The tag persistence was checked daily and moulted crayfish were retagged.

### Data collecting

Our experiment was performed in the first half of November 2011. Over two weeks we video-recorded dyadic encounters in each crayfish group for one hour once in days 1, 4, 7, 10, and 13 post-grouping, starting at 16:00 when the locomotion activity of the red swamp crayfish had been found to be highest at the given temperature^35^. For video-recording, we used five digital camcorders (Camileo S20, Toshiba, Tokyo, Japan) mounted on tripods placed vertically above the observation tanks.

### Data analysis

Each interaction was formed by two individuals: an initiator (an individual who approached the other one) and a recipient (the approached individual). During each encounter, we recorded the Type of agonistic activity: (i) whether the initiator aggressively charged the recipient frontally and grabbed it with the chelae (further referred as Attack); (ii) whether the initiator aggressively charged the recipient from behind (further referred as Harassment); (iii) whether the recipient defended itself by its chelae against aggressive physical contact provoked by the initiator, the encounter was assessed as a Fight; or (iv) just approached the opponent, which did not lead to contact but did evoke a response (further referred as Approach). Based on the result of the encounter, the retreating individual was considered the ‘loser’ if it had separated from the winner by a distance of more than two total body lengths. Its opponent was deemed the ‘winner’. The end of the encounter was considered when the loser stopped responding. If both the initiator and recipient withdrew, the encounter resulted in a ‘draw’. We also recorded how the loser behaved in response to the aggressive encounter, e.g. retreated backwards or escaped using the retrograde escape response (tail flip). If the recipient defended itself by chelae against aggressive physical contact provoked by the initiator, the encounter was assessed as a Fight. The Fight was an only type of encounter running for a period of time; other types were simply ended by retreat or escape of the loser immediately after initiator started the encounter.

If the individual lost one or both chelae, it was excluded from the analysis. We also excluded complete groups where one or more members died or where two or more individuals lost their tags at the same time and their unambiguous retagging was therefore impossible. Furthermore, we excluded from analysis all non-aggressive interactions such as approach without physical contact between initiator and recipient.

### Statistical analysis

Associations between total numbers of agonistic encounters and their distribution in time, the ratio of agonistic encounters among Group types, and agonistic encounters within each Group type were tested using a multivariate General Linear Mixed Model (GLMM, PROC MIXED, SAS, version 9.4), with total numbers of agonistic encounters as a dependent variable (Tables S1 and S2). To account for the use of repeated measures on the same group, the analysis was performed using mixed model analysis with the ID of an individual nested within the group as a random factor. The effects were one continuous variable and classes. The continuous variable was Time (1 to 13 days). Classes were Type of agonistic activity (Attack, Fight, and Harassment) and Group type (MI, ES, Mix). An interaction between Type of agonistic activity*Group type was also added. Least-squares means (LSMEAN) were computed for each class and differences between classes were tested by t-test. We used a Tukey-Kramer adjustment for multiple comparisons. Associations between the dependent variable and Time was estimated by fitting a random coefficient model using PROC MIXED as described by Tao *et al*.^36^. We calculated predicted values of the dependent variable and plotted them against the fixed effect with predicted regression lines for each group. Where more than one value was plotted in the same position of the chart, we used a bubble type of plot.

## Supplementary information

Dataset 1
