# Supplementary material for: Early ontogeny social deprivation modifies future agonistic behaviour in crayfish
Source: Sci Rep. 2019 Mar 20;9:4667. doi: 10.1038/s41598-019-41333-8 (PMC6427012; doi:10.1038/s41598-019-41333-8)
Supplement: Supplementary file 1 — Dataset 1 [file 41598_2019_41333_MOESM1_ESM.pdf]

Early ontogeny social deprivation modifies future agonistic behaviour in crayfish

Jiří Patoka, Lukáš Kalous, Luděk Bartoš

Table S1. Total numbers of agonistic encounters according day and group type (ES = early separated, MI = maternally incubated, Mix = mixed group).

Day	Group type	N
1	ES	447
1	MI	332
1	Mix	302
4	ES	686
4	MI	665
4	Mix	499
7	ES	537
7	MI	488
7	Mix	367
10	ES	642
10	MI	472
10	Mix	355
13	ES	486
13	MI	632
13	Mix	326
	SUM	**7236**

Table S2. Total numbers of agonistic encounters according type encounter (A = attack, F = fight, H = harassment) and group type (ES = early separated, MI = maternally incubated, Mix = mixed group).

**Encounter**	**Group type**	**N**
A	ES	890
A	MI	1080
A	Mix	511
F	ES	669
F	MI	559
F	Mix	459
H	ES	1239
H	MI	950
H	Mix	879
	SUM	**7236**
